# Challenging diagnosis of male intraductal papilloma masquerading as eccrine hidradenoma in the breast: Case report

**DOI:** 10.1097/MD.0000000000037607

**Published:** 2024-03-29

**Authors:** Xinyue Liu, Jie Du, Lirong Zhao

**Affiliations:** a Ultrasound Diagnostic Center, The First Hospital Affiliated to Jilin University, Changchun, Jilin, China.

**Keywords:** diagnostic challenges, histopathological analysis, intraductal mammary papilloma, misdiagnosed breast lesion, ultrasound pitfalls

## Abstract

**Rationale:**

This article presents a challenging case involving an elderly male patient with a misdiagnosed intraductal mammary papilloma initially identified as a sweat adenoma through ultrasound imaging. The study aims to explore the histopathology, clinical presentations, and sonographic features of both conditions, emphasizing the contributing factors to the diagnostic misstep.

**Patient Concerns:**

A 61-year-old male reported a persistent left breast mass, along with pain and swelling, spanning a 6-month duration.

**Diagnoses:**

Ultrasound examination indicated a deep, square, mixed-echo mass in the left nipple, initially suggestive of a sweat adenoma. However, subsequent pathological analysis following resection under general anesthesia confirmed an intraductal papilloma.

**Intervention:**

The patient underwent surgical resection of the left breast mass under general anesthesia.

**Outcome:**

Post-surgery, the patient exhibited satisfactory recovery; however, regrettably, he was lost to follow-up.

**Lessons:**

This study underscores the challenge in differentiating between clear cell sweat adenoma and male intraductal mammary papilloma solely based on ultrasonic characteristics. It emphasizes the susceptibility of ultrasound-based diagnoses to misinterpretation, highlighting the critical need for a comprehensive pathological examination to establish a definitive diagnosis.

## 1. Introduction

Intraductal papilloma in the male breast is a noncancerous lesion. It commonly presents with unilateral nipple blood or serous discharge as its typical clinical manifestation. On ultrasound, a characteristic finding is catheter dilation accompanied by luminal solid nodules. It is noteworthy that male intraductal papilloma carries an elevated risk of both cancer and recurrence. Some experts posit that it could potentially serve as an early indicator of invasive cancer.^[1]^ Consequently, adequate clinical attention should be devoted to diagnosing and managing intraductal papilloma in the male breast.

## 2. Case report

**Figure 1.**
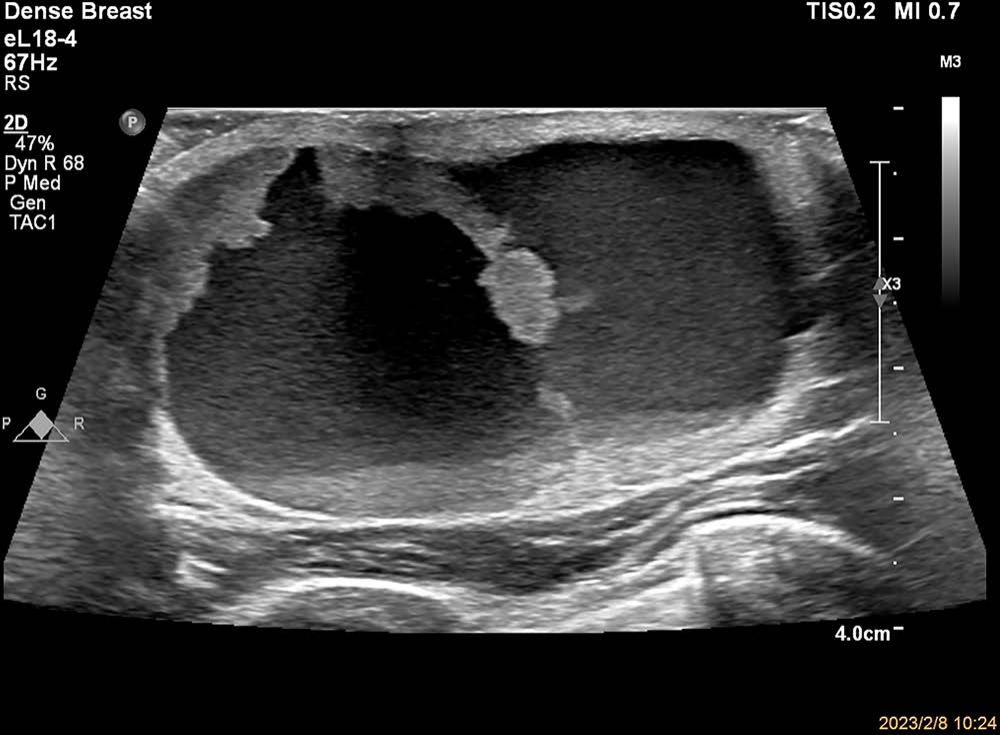
Male, 61 years old, intraductal papilloma of the breast, gray-scale ultrasound showing subcutaneous mixed echogenic mass.

**Figure 2.**
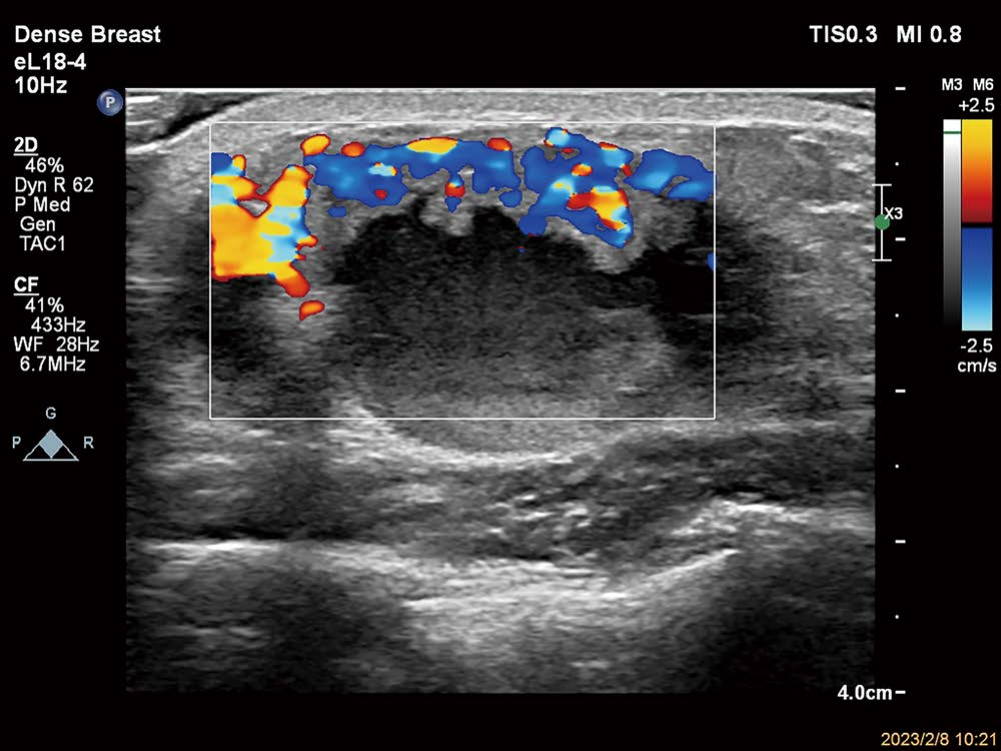
Color Doppler flow imaging (CDFI) showing abundant blood flow signals in the solid echogenic portion. CDFI = color doppler flow imaging.

A 61-year-old male patient was hospitalized after the identification of a left breast mass that had been present for the last 6 months. Initially noticed 6 months ago, the patient observed a left breast mass roughly the size of an egg. Without seeking systematic treatment initially, the patient reported recent developments, including increased pain and a noticeable enlargement of the mass. Notably, there was no history of nipple discharge. During the physical examination, asymmetry between the breasts was noted, with the left breast displaying surface elevation and dark red coloration. Palpation identified a palpable mass on the left, measuring approximately 6.0 cm × 5.0 cm. The mass exhibited a soft texture, tenderness, and a fluctuant sensation, yet no nipple discharge was observed. The breast ultrasound exhibited a predominantly cystic mixed echogenic mass located approximately 2 mm beneath the skin at the deep square distance of the left nipple. Measuring 52 mm × 50 mm × 29 mm with clear borders, the mass displayed irregular morphology, featuring hyperechoic areas within the cystic portion and visible septations. The solid component was irregular, and its echogenicity appeared relatively homogeneous (Fig. 1). Color Doppler ultrasound revealed relatively abundant blood flow signals at the boundary between the cystic and solid portions (Fig. 2). The ultrasound diagnosis identified the mass as a mixed echogenic mass in the depth of the left nipple, with eccrine hidradenoma not ruled out. Clinically, the diagnosis was documented as an “occupying lesion of the breast,” prompting the patient to undergo general anesthesia for the surgical excision of the left breast mass.

During the surgery, a meticulous layer-by-layer dissection of the skin and subcutaneous tissue revealed a cystic mass. This mass was entirely removed, and upon incision, a flow of dark red, bloody fluid was observed. Further examination of the mass exposed an irregular solid nodule, approximately 2 cm × 1 cm in size. A rapid frozen section analysis conducted intraoperatively confirmed the diagnosis of intraductal papilloma with active epithelial proliferation. The postoperative pathology report described the papillary proliferation of ductal epithelium and localized active ductal epithelial proliferation with atypical hyperplasia. Additionally, portions of the cyst wall showed evidence of previous hemorrhage and crystallized cholesterol deposits. The patient’s postoperative recovery was uneventful. However, notably, the patient was subsequently lost to follow-up.

## 3. Discussion

In the normal physiology of adult males, the breast is predominantly composed of adipose tissue, featuring a limited presence of atrophic ducts and stromal components. In normal adult males, the breast primarily consists of adipose tissue, with only a small amount of atrophic ducts and stromal components.^[2]^ Consequently, ductal and stromal lesions are exceedingly rare in the male breast,^[3]^ and male intraductal papillomas are even more infrequent. The typical clinical presentation of male intraductal papillomas includes unilateral bloody or serous nipple discharge. Sonographically, intraductal papillomas exhibit characteristic features such as ductal dilation with intraductal solid nodules, occasionally accompanied by fine punctate echoes within the dilated ducts. In certain instances, they may present as a complex cystic and solid mass called intracystic papilloma.^[4]^ Male Intraductal papilloma is a noncancerous lesion that carries an elevated risk of both cancer and recurrence. Some experts posit that it could potentially serve as an early indicator of invasive cancer.^[1]^ Therefore, male intraductal papillomas should receive adequate clinical attention.

Hidradenomas are classified based on their histological types and differentiation grade, including eccrine poroma, syringoma, and clear cell hidradenoma (CCH). Notably, CCH is the most prevalent subtype^[5]^ and is regarded as a benign tumor originating from the eccrine sweat glands.^[6]^ CCH can also exhibit malignant forms. However, research has indicated that malignant CCH does not arise from the benign subtype.^[7]^ The ultrasound appearance of CCH can exhibit variations influenced by factors such as growth rate, vascularity, mucinous material proportion, and internal hemorrhage. Typically, CCH can be categorized into 3 types: cystic and solid mixed-type, solid mass type, and thick-walled cystic type.^[8]^ CCH lesions may display diverse surface colors, including red, blue, normal skin color, and brown. Although CCH primarily manifests in the skin of the scalp, face, anterior trunk, and limbs, literature reports suggest its occurrence in the breast as well.^[8,9]^ Breast CCH is rare, and some studies have indicated that it may coexist with both eccrine sweat glands and mammary ducts.^[10]^ When CCH is situated within the mammary duct, its ultrasound appearance manifests as a cystic lesion within the deep breast tissue. In such cases, the lesion is no longer confined to the skin layers, posing challenges in distinguishing it from intraductal papillomas on ultrasound images. When CCH develops within the mammary duct, its ultrasound presentation appears as a cystic lesion deep within the breast tissue. In these instances, the lesion is situated beyond the skin layers, posing a challenge in distinguishing it from intraductal papillomas on ultrasound images.

The primary factor contributing to the misdiagnosis in this case analysis stems from the patient being male, presenting with a breast lump, and lacking a history of nipple discharge. The physical examination revealed that the lump displayed a dark red skin color; ultrasound revealed a mixed echoic mass with the nipple deep and closely adjacent to the skin, resembling a cystic and solid mixed-type hidradenoma. Moreover, the limited awareness among physicians regarding male intraductal papillomas led to higher initial consideration of the possibility of a hidradenoma.

This passage outlines vital factors in differentiating between intraductal papilloma and breast CCH. First, the patient’s history merits close examination, particularly for any record of bloody or mucinous nipple discharge. Additionally, meticulous attention to the color of the lesion’s skin surface is critical, focusing on identifying any red, blue, or brown discolorations. In this case, the lesion exhibited a dark red hue, suggesting possible internal hemorrhaging within the lesion.

Nevertheless, it is imperative to acknowledge the limitations and challenges encountered in this diagnostic process. The scarcity of male intraductal papillomas and breast CCH, coupled with their shared ultrasound features, inherently contributes to the difficulty in differentiation. The reliance on ultrasound alone may lead to misinterpretation, as demonstrated in this case. Additionally, the lack of a comprehensive follow-up due to the patient being lost to follow-up impedes a thorough understanding of long-term outcomes and potential complications. These limitations underscore the need for continued research, improved diagnostic modalities, and heightened clinical awareness to enhance accuracy in distinguishing between these rare entities.

## 4. Conclusion

In conclusion, differentiating between male intraductal papillomas and CCH via ultrasound poses challenges, highlighting the necessity for a definitive pathological diagnosis. The rarity of these lesions in the male breast, coupled with overlapping sonographic features, emphasizes the importance of a meticulous patient history and clinical symptom assessment. The case underscores the need for increased awareness among physicians to avoid misdiagnosis, ensuring accurate characterization and appropriate management of these distinct entities.

## Acknowledgments

We thank *Medjaden Inc.* for its assistance in the preparation of this manuscript.

## Author contributions

**Conceptualization:** Lirong Zhao.

**Data curation:** Xinyue Liu.

**Supervision:** Lirong Zhao.

**Writing – original draft:** Xinyue Liu.

**Writing – review & editing:** Jie Du, Lirong Zhao.
